# Differentiated memory and scientific cognition in AI research agents

**DOI:** 10.3389/fdata.2026.1916523

**Published:** 2026-08-05

**Authors:** Diego F. Cuadros, Abdoul-Aziz Maiga, Sid Thatham, Alvaro Ortiz, Margaret Powers-Fletcher, Ming Tang

**Affiliations:** 1 Digital Epidemiology Laboratory, Digital Futures, University of Cincinnati, Cincinnati, OH, United States; 2 Department of Biological Sciences, University of Cincinnati, Cincinnati, OH, United States; 3 Center for Humanities and Technology (CHaT), University of Cincinnati, Cincinnati, OH, United States; 4 Norwegian University of Science and Technology, Trondheim, Norway; 5 UC Utilities, University of Cincinnati, Cincinnati, OH, United States; 6 Department of Mathematical Sciences, University of Cincinnati, Cincinnati, OH, United States; 7 College of Arts and Sciences, University of Cincinnati, Cincinnati, OH, United States; 8 School of Architecture and Interior Design, University of Cincinnati, Cincinnati, OH, United States; 9 eXtended Reality (XR) Laboratory, Digital Futures, University of Cincinnati, Cincinnati, OH, United States

**Keywords:** AI for science, AI research agents, cognitive architecture, differentiated memory, large language model agents, provenance, scientific cognition, scientific judgment

## Abstract

AI research agents increasingly support ideation, literature search, coding, experimental execution, analysis, and manuscript drafting across the scientific workflow. This progress advances automated discovery, but workflow automation is not scientific cognition. Scientific reasoning is cumulative and path-dependent: it depends on what a researcher has written, read, learned from critique, absorbed through experience, and used as habitual standards for judging novelty, rigor, feasibility, and significance. We propose Mnemo as a framework for modeling scientific cognition in AI research agents. First, scientific cognition may require differentiated memory, organized into distinct spaces for authored work, external reference, critique, experience, and judgment. Second, provenance should be treated not as passive metadata but as memory routing, because source origin helps determine cognitive function in reasoning. Third, new ideas may be better modeled as controlled collisions across memory spaces, filtered by judgment, rejection, and epistemic calibration, than as generic recombination from model priors. Mnemo motivates a research agenda for AI in science centered on routing fidelity, critique use, judgment alignment, rejection quality, and epistemic calibration.

## 1 Introduction: the automation-cognition gap

AI systems are beginning to automate large portions of scientific work, extending a long-standing ambition to automate science (Waltz and Buchanan, 2009). Recent research-agent frameworks can search the literature, propose hypotheses, write and debug code, run experiments, analyze outputs, and draft manuscripts. Lu et al. (2026) extended this trajectory with The AI Scientist, an end-to-end system for automated machine-learning research. They reported that, under a pre-specified study protocol, one AI-generated workshop manuscript received scores sufficient for first-round acceptance before being withdrawn according to protocol. The result is a meaningful milestone: many observable stages of computational research can now be decomposed into tasks that language models and agentic orchestration can execute with growing competence.

Workflow automation, however, is not scientific cognition. A system may complete many observable tasks of research without representing the memory structure that makes scientific reasoning cumulative, selective, and generative. Many current research agents remain weakly grounded in longitudinal intellectual history: they do not clearly distinguish between what a researcher authored, what they studied or were influenced by, what external critique revealed about the limits of prior arguments, or how accumulated experience shaped later choices. Nor do they formalize judgment as a separate cognitive layer. The result is a deeper architectural gap. Systems built mainly to sequence tasks may produce plausible outputs, but they lack an account of how inquiry is routed through memory, pressure-tested by critique, and filtered by standards of significance, feasibility, and rigor.

In this Perspective, we argue that the next methodological challenge for AI in science is not only better automation but a cognitive architecture for research reasoning. We propose Mnemo as a framework built around differentiated memory, provenance-based routing, cross-memory idea generation, and an explicit judgment layer. The central hypothesis is that scientific novelty does not emerge from flat retrieval or generic ideation alone, but may instead arise from structured interactions among authored work, external reference, critique, experience, and judgment. On this view, a reviewer objection can become the seed of the next research question, just as a method developed in one project can be reframed for a different problem encountered years later. Provenance determines what kind of memory a system is using in a given inference. Mnemo is not presented as a finished autonomous scientist, but as a research agenda for building and evaluating AI systems grounded in intellectual continuity rather than workflow completion alone.

Formative implementation experience sharpens this claim without turning the Perspective into a system report. Early internal prototyping suggests that differentiated memory and typed collisions may help surface non-trivial, grounded candidate directions, but also that collisions alone are insufficient. The strongest outputs appear to depend on explicit judgment, rejection, and epistemic calibration, especially when the system must distinguish strong structural framings from merely attractive ones. These lessons do not change the conceptual argument of this Perspective; they clarify where the most difficult bottlenecks lie.

## 2 Why workflow automation is not scientific cognition

Recent progress in AI research-agent systems has been organized largely around workflow coverage: which stages of the research process can be automated, how reliably, and with what degree of human oversight. This framing has been productive. It has yielded systems that can search the literature, generate hypotheses, write and execute experimental code, interpret results, and produce manuscript drafts, sometimes within a single loop. The AI Scientist showed that such a pipeline can, under controlled conditions, produce work that passed first-round peer review at a workshop venue before being withdrawn under the study protocol (Lu et al., 2026). Similar workflow-oriented agentic systems are now being discussed across biomedical discovery, healthcare research, and economics (Gao et al., 2024; Korinek, 2025; Njei et al., 2026).

Work on memory-augmented language agents already recognizes that agent competence depends on more than a fixed context window. Retrieval-augmented generation improves access to external knowledge (Lewis et al., 2020), MemGPT treats memory management as a system-level problem for long-running agents (Packer et al., 2023), CoALA organizes language agents around modular memory, action space, and decision-making procedures (Sumers et al., 2024), and recent surveys and systems describe increasingly sophisticated mechanisms for storing, consolidating, and retrieving agent memories (Zhang et al., 2024; Xu et al., 2025). Mnemo builds from this literature but asks a narrower question: not whether agents can store information or persist across sessions, but what memory distinctions are needed for sustained scientific reasoning over a researcher's longitudinal intellectual history.

Workflow coverage, however, is an engineering metric, not a cognitive one. A system can execute many stages of research without representing the memory structure that makes scientific reasoning cumulative, selective, and generative. It may search, write, analyze, and revise while lacking an explicit model of why one question is worth pursuing, why a critique should reshape an argument, or why a method that succeeded in one context may fail in another. The central limitation of current research agents is therefore not simply imperfect task execution, but the absence of an architecture for sustained scientific thinking.

The gap becomes clearest in three domains: memory, critique, and judgment. First, many current research agents treat retrieved material as prompt context rather than as differentiated memory. Even when systems use retrieval, literature search, or short-horizon logs, the resulting information is rarely organized into enduring memory spaces with distinct provenance and cognitive roles. A passage from a researcher's own paper, an external reference, and a reviewer's critique may all become text in context, but they do not function equivalently in scientific reasoning. The distinction between what I wrote, what I studied, and what others said about my work changes the evidentiary status of a memory and the inferences it should support. Flattening these distinctions into a single retrieval pool removes the structure that makes scientific memory cumulative rather than merely additive.

Second, current systems generally treat critique as a filter rather than as a cognitive resource. In systems that incorporate peer review or evaluation, critique usually appears after generation as a score, a gate, or a revision signal. But a serious reviewer objection does more than mark weakness; it can reveal where an argument is most exposed and where the next productive question may lie. Many research directions begin not with an empty space in the literature, but with a point where an earlier claim broke under external pressure. Systems that use critique only for *post hoc* evaluation cannot use it as a source of generative insight.

Third, current research agents lack an explicit judgment layer. Ideas may be generated from model priors, retrieved literature, or experimental logs, but generation is not judgment. Scientific judgment involves weighing feasibility against novelty, significance against execution cost, and short-term opportunity against long-term trajectory coherence. These tradeoffs are shaped by a researcher's history, disciplinary norms, funding constraints, and accumulated experience of what reviewers, editors, and funders actually reward. A system without a judgment layer can produce many candidate ideas but cannot reliably distinguish the few worth pursuing from the many that are technically possible but scientifically weak or strategically misaligned. In this respect, the limitations reported by Lu et al. are instructive: generated ideas were often described as “naive or underdeveloped” (Lu et al., 2026). That weakness reflects a deeper architectural problem: idea generation without grounded judgment.

Together, these limitations suggest that the challenge is not simply to automate more of the research pipeline, but to formalize the memory structure and evaluative processes that make scientific reasoning cumulative, self-correcting, and generative. The next sections develop that claim through differentiated memory, provenance-based routing, and judgment-guided cross-memory collisions.

## 3 Differentiated memory as the architecture of research intelligence

If workflow automation is not enough to model scientific cognition, the next question is what architecture is missing. Our proposal is that scientific reasoning may depend on differentiated memory rather than a single undifferentiated store. Scientists do not think from a flat archive. They reason through multiple kinds of memory that differ in provenance, authority, function, and consequence. What they wrote serves a different role from what they read. What others said about their work serves a different role again. Experience acquired through teaching, mentoring, and other explanatory or practice-facing roles may not appear in a publication record, yet it still shapes how problems are framed and prioritized. For an AI agent, this does not mean that experience is already present in model pre-training. It means that practice-facing traces, interactions, decisions, and outcomes must be externalized, attributed, and made available as inspectable memory. Over time, recurrent patterns of choice and rejection become a distinct form of memory in their own right: not memory of content, but memory of judgment.

This distinction is easy to obscure in AI systems because most machine representations reduce memory to stored text plus retrieval. Scientific memory is not just a larger context window. It is a structured system in which different memory spaces support different forms of reasoning. The key question is therefore not simply which documents are relevant, but what kind of memory a system should be thinking with for a given task.

We propose five memory spaces as a candidate minimal operational architecture for scientific cognition. **Core Memory** contains the researcher's own intellectual work: papers, drafts, grants, code, planning notes, and responses to critique. It provides continuity of identity and the main substrate for recall and trajectory reconstruction. **Reference Memory** contains external work the researcher has read or used. It provides context, precedent, and conceptual adjacency, but it is not identical to the researcher's own position. **Critique Memory** contains reviewer comments, editor decisions, collaborator critiques, and other external evaluations. It records not only what was challenged, but where arguments proved most vulnerable.

**Figure 1:**
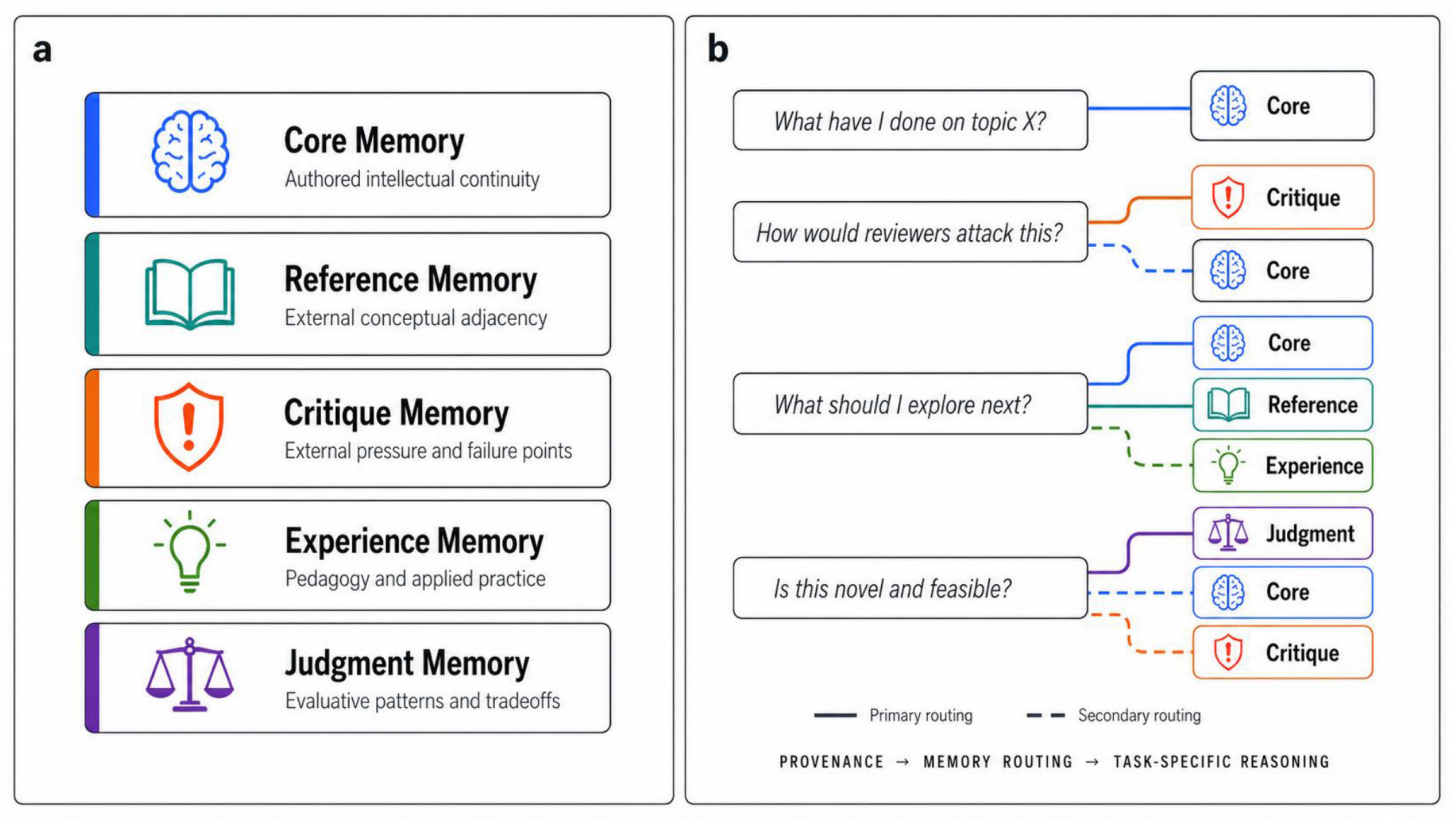
Differentiated memory and task-dependent routing. **(a)** Mnemo partitions scientific cognition into five memory spaces with distinct roles: Core (authored intellectual continuity), Reference (external conceptual adjacency), Critique (review pressure and failure points), Experience (pedagogical and mentoring practice), and Judgment (evaluative patterns for strategy and feasibility). **(b)** the same query type does not route to a flat corpus; each task activates a different primary and secondary memory configuration.

Experience Memory contains pedagogical, mentoring, and explanatory practice material. Although often excluded from research-centered accounts of cognition, these materials shape how researchers explain concepts, recognize misunderstandings, and scope feasible work. Administrative or service documents belong in this space only when they directly expose explanatory failures, operational constraints, or researcher-facing demands that can reshape inquiry. Operationally, Experience Memory would be populated from concrete and largely conventional artifacts: lecture materials and their revision histories, course syllabi, examination questions and anonymized performance patterns, question-and-answer logs from teaching platforms, office-hours and mentoring notes, thesis-committee feedback, and, where consent permits, transcripts of lab meetings and recorded lectures. Recurring student misunderstandings can be captured as structured annotations on the concepts that produce them. This is the most capture-limited of the five spaces: much explanatory practice is oral, contextual, and never recorded, and consent and privacy constraints properly bound what can be externalized. The claim is not that experience can be captured exhaustively, but that even partial, consented traces of explanatory practice carry cognitive signal that formal research outputs do not. Judgment Memory is an extracted layer of procedural and evaluative patterns rather than a raw document store. It captures how a researcher chooses methods, frames significance, rejects weak ideas, responds to criticism, and sequences priorities. Whether these five spaces are the minimal sufficient set is an empirical question, but they capture the distinctions most directly implicated in how sustained research reasoning differs from single-session retrieval. Figure 1 shows these five memory spaces and the task-dependent routing they support.

Classical cognitive architectures such as Soar and ACT-R long emphasized specialized memory and control systems (Laird et al., 1987; Anderson et al., 2004), and contemporary language-agent frameworks similarly distinguish working memory, long-term memory, internal actions, and external actions (Sumers et al., 2024). The novelty of the present framework is not modularity alone, but the claim that scientific reasoning may depend specifically on longitudinal, provenance-sensitive distinctions among authored work, external influence, critique, experience, and judgment.

These five spaces should therefore not be read as a replacement for broad cognitive categories such as working, episodic, semantic, or procedural memory. They cut across those categories. A researcher's manuscript, a cited reference, and a reviewer's criticism may all concern the same semantic topic and may belong to the same episode of work, but they license different inferences because they occupy different epistemic roles. Mnemo's contribution is to make those scientific roles first-class routing variables.

Getting these memory spaces right is a pre-requisite for any meaningful routing. Before a system can reason over an archive, it must know what kind of intellectual object it is handling and what relationship that object bears to the researcher's work. Provenance classification is therefore not merely housekeeping. If authorship is assigned incorrectly, the system may mistake external work for the researcher's own prior reasoning and generate future ideas from the wrong intellectual base. Artifact identity matters for the same reason: scientific cognition unfolds over canonical works, not arbitrary files. Drafts, publications, reviewer comments, and responses may belong to a single intellectual episode while still occupying different memory spaces and serving different cognitive functions.

The importance of these distinctions is not taxonomic but functional. A paragraph from a researcher's own manuscript, a methodological claim from an external reference, and a reviewer's criticism of that same manuscript may all concern the same topic, but they cannot be treated as interchangeable evidence. They carry different inferential permissions: different constraints on what conclusions can be drawn from them and what reasoning they should license. The authored text expresses prior commitment. The external reference provides context or influence. The critique identifies weakness or unresolved pressure. In this sense, provenance functions as memory routing.

Once memory is differentiated in this way, routing becomes the mechanism that turns storage into cognition. A system should not query all memory spaces equally for every task. “What have I done on topic X?” should route mainly to Core Memory. “What literature shaped this line of work?” requires Core and Reference Memory together. “How would reviewers attack this argument?” should route first to Critique Memory. Teaching or explanation tasks should preferentially engage Experience Memory. Strategic questions such as “Is this novel enough but still feasible?” should draw on Judgment Memory as well. Routing is not only a retrieval convenience. It is the mechanism by which different forms of memory constrain different forms of inference. In each case, the central problem is not relevance ranking alone, but identifying the right memory configuration for the task.

Differentiated memory is what allows scientific reasoning to become cumulative rather than merely additive. A flat archive can retrieve similar text. A differentiated memory architecture can distinguish continuity from influence, objection from endorsement, and experience from explicit argument. It can preserve the fact that a reviewer's criticism belongs to the same intellectual episode as a manuscript while still recognizing that the criticism should not be treated as authored belief. Without these distinctions, an AI system may still produce useful outputs, but it will do so without an internal model of how scientific thought is organized over time.

Differentiated memory is therefore not just a filing strategy layered onto an otherwise standard research agent, but a claim about the architecture of research intelligence itself. Once these memory spaces are made explicit, a further question becomes possible: how do they interact to generate new ideas rather than merely better retrieval? The next section addresses that question.

## 4 Cross-memory collisions as the mechanism of idea generation

Once memory has been differentiated, a further question becomes unavoidable: how might these memory spaces generate new ideas rather than merely support better retrieval? Our proposal is that scientific novelty may often be better modeled as controlled collisions between memory spaces than as random recombination or one-shot ideation from model priors. We define a cross-memory collision as a structured interaction between records from two or more memory spaces with different provenance, evaluated for the tensions, transfers, or reframings it produces. The point is not simply that multiple sources are combined. It is that the kinds of memory being combined matter. In this Perspective, collisions should be understood as a plausible minimal testable generative mechanism rather than as a complete theory of scientific cognition.

The most intuitive collision operator is **Core x Reference**. This is the encounter between what the researcher has already done and what exists just beyond that prior work. It can take several forms: a method from the archive applied to an external problem, an external method applied to a problem already present in the archive, or a reference paper reframed through the researcher's established trajectory. Novelty here comes from bringing continuity into contact with adjacency. Because one side of the collision is anchored in authored work and the other in external influence, the resulting idea is not only new in the abstract. It is newly plausible for that particular researcher.

A second operator, **Core x Core (temporal)**, captures a form of novelty that flat retrieval systems are likely to miss. Researchers do not simply accumulate publications; they evolve through them. Older methods may become newly relevant when new datasets or framings appear, and recent conceptual lenses may illuminate earlier work in new ways. Temporal collisions treat a research career as a dynamic intellectual system rather than a static portfolio. Novelty here arises not from external importation but from reactivating latent continuity within the archive itself.

A third and particularly important operator is **Core x Critique**. Scientific novelty is often portrayed as the discovery of a gap in the literature, but many productive frontiers originate where a prior argument failed under pressure. A reviewer objection, an editor's concern, or a failed rebuttal can reveal not only weakness but direction. A criticism that once blocked publication may, under a different framing, become the seed of the next research question. For example, a reviewer may dismiss a spatial analysis as merely descriptive; that criticism can become the next question by forcing the researcher to ask whether the observed geography reflects shared structural determinants across diseases or genuinely disease-specific mechanisms. If critique is treated only as a *post hoc* evaluation signal, this generative role disappears. **Core x Critique** formalizes the ability to convert external pressure into new inquiry.

A related operator, **Reference x Critique**, captures a different kind of frontier. Sometimes the literature appears to suggest a direction, but accumulated critique reveals why that direction remains unstable or incomplete. Here the productive idea emerges from the mismatch between published possibility and experienced resistance. Some frontiers are found not by locating empty spaces in the literature, but by examining where apparently available paths have repeatedly broken down.

The operator **Experience x Core** introduces a further source of grounded novelty that conventional research pipelines typically ignore. Teaching, mentoring, and other explanatory or practice-facing encounters often expose recurring misunderstandings, hidden constraints, and practical bottlenecks that do not appear in papers alone. A concept that students repeatedly struggle to grasp may indicate a deeper conceptual ambiguity, and a mentoring discussion may reveal a tractable problem that an expert had normalized and stopped noticing. In some cases, an explanatory strategy developed in teaching can itself become a research insight. Novelty here emerges from contact between formal research memory and the settings in which research is taught, translated, and applied.

**Figure 2:**
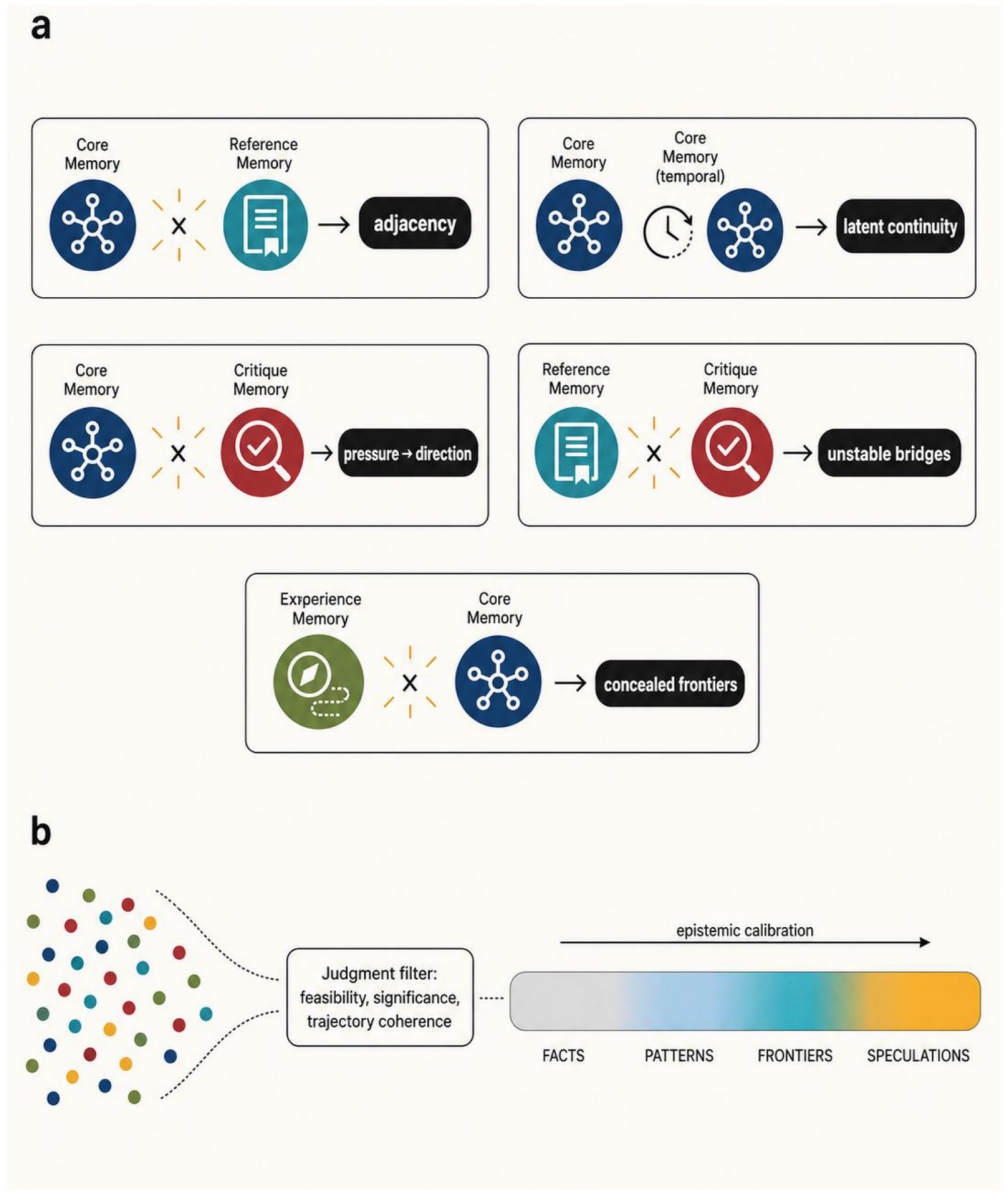
Cross-memory collisions as the generative mechanism. **(a)** Mnemo models idea generation as typed collisions between memory spaces with different provenance (Core x Reference, Core x Core-temporal, Core x Critique, Reference x Critique, Experience x Core), each associated with a characteristic novelty signature. **(b)** candidate frontiers pass through a judgment filter before being expressed along an epistemic gradient from facts to patterns, frontiers, and speculations.

These operators are not interchangeable, because provenance constrains the kind of novelty they can produce. Core x Reference imports adjacency. Core x Core (temporal) recovers latent continuity. Core x Critique transforms pressure into direction. Reference x Critique identifies unstable bridges. Experience x Core surfaces conceptual or practical frontiers that formal outputs alone may conceal. Cross-memory collisions are therefore more than a metaphor for recombination. They are typed operations over memory spaces with different epistemic roles. Provenance shapes not only retrieval, but the form of novelty a system is allowed to propose. Figure 2 summarizes these operators and their characteristic signatures.

Because collision-based generation moves from evidence toward proposal, it also requires explicit epistemic calibration. A system should not present every cross-memory association as a valid research direction. Outputs should remain legible along a fact-to-speculation gradient: **facts** about what the archive contains, **patterns** across that archive, **frontiers** suggested by typed collisions, and **speculations** about what should be pursued next. A useful system must also be able to identify strong but unsupported extrapolations and explicitly state what a proposed framing does not yet justify. This gradient matters because the value of a generative system depends not only on novelty, but on traceability.

One concrete mechanism for enforcing this gradient is evidence-linked claim gating. Every candidate frontier generated by a collision must carry typed citations into the memory spaces that produced it, the specific Core records, Reference materials, or Critique entries on which it rests. A separate verification pass, implemented as a distinct prompt or model call that does not share the generator's context, then performs three operations. It enumerates the cited records and confirms that they support the claim as stated; it actively searches the memory spaces for disconfirming records; and it assigns the claim a tier on the fact-to-speculation gradient according to an explicit rubric (for example, direct textual support in two or more spaces; support in one space with no disconfirmation found; suggestive adjacency only). Claims whose support falls below a set threshold, fewer than a minimum number of independent supporting records, or any unresolved disconfirming record, are automatically downgraded to speculation or withheld with a stated reason. The thresholds themselves are evaluable parameters, raising them should trade generative volume against precision in measurable ways, which is what makes overclaim control a testable property of the system rather than a stylistic aspiration.

Cross-memory collisions, then, provide a plausible minimal mechanism by which differentiated memory becomes generative. They explain how a system might move from structured recall to grounded candidate novelty without collapsing into either flat retrieval or unconstrained ideation. But collision alone is not enough. Many possible collisions will be trivial, weakly grounded, strategically mistimed, or simply overframed. What matters is not only that collisions occur, but that they can be filtered, rejected, or deferred for defensible reasons. If differentiated memory helps explain where candidate ideas come from, a separate judgment layer is needed to explain which of those candidates deserve to survive.

## 5 Judgment as a separate cognitive layer

If cross-memory collisions explain where candidate ideas come from, they do not explain why only a few should survive. Collision alone produces possibility; science requires selection. This is why judgment must be treated as a separate cognitive layer rather than as a by-product of retrieval or generation. A system may generate many plausible directions by combining memory spaces in interesting ways, but without an explicit mechanism for prioritizing, rejecting, postponing, or reframing those directions, it remains a combinatorial engine rather than a model of scientific reasoning.

Judgment in this sense is not reducible to a single score or preference function. It is an organized pattern of tradeoffs. Researchers weigh novelty against feasibility, significance against execution cost, strategic timing against intellectual curiosity, and short-term opportunity against long-term trajectory coherence. They learn these tradeoffs over time through reviewer objections, failed submissions, abandoned projects, funder priorities, student capacity, and prior methodological successes. Judgment is therefore not simply a matter of taste. It is a historically accumulated filter on possible futures, consistent with longstanding accounts of tacit knowledge and disciplinary paradigms in science (Polanyi, 2009; Kuhn, 2012).

This is why we treat **Judgment Memory** as distinct from the other memory spaces. It is not a raw document store, but an extracted layer of procedural and evaluative patterns: how a researcher scopes a feasible study, what kinds of novelty they consider meaningful, when they decide that an idea is too diffuse or too incremental, how they respond to criticism, and how they rank competing directions. Put differently, Judgment Memory is the memory of how choices are made. This is also why Critique Memory feeds directly into judgment formation: accumulated external pressure does not merely test ideas after the fact, but shapes the criteria by which future ideas are evaluated before they are fully formed.

In practice, Judgment Memory would be constructed from paired archival traces and direct elicitation rather than inferred from prose alone. Reviewer responses and grant revisions show which criticisms changed claims, scope, or significance arguments. Draft-to-final diffs and planning notes reveal how ideas were reframed, postponed, or abandoned. Ranking tasks over plausible frontiers can elicit explicit preference pairs and rationales, while rejection benchmarks can test whether the resulting judgment layer rules out weak ideas for the right reasons. These sources do not exhaust judgment, but they make it operational enough to extract, audit, and improve.

These negative traces matter for a further reason. They are systematically underrepresented in the corpora on which foundation models are trained. The published literature preserves successes, while failed proofs, rejected framings, and abandoned directions rarely enter the public record, so model priors encode a survivorship bias toward ideas that worked. A researcher's archive is one of the few places where the negative space of inquiry is recorded at all, in rejected drafts, unfunded proposals, reviewer objections that ended a line of work, and directions quietly dropped. Externalizing these traces into Critique and Judgment Memory therefore gives a Mnemo-based agent access to precisely the class of evidence that pre-training cannot supply, and on which the distinction between promising and merely plausible ideas most depends.

This distinction is important because an AI judgment layer cannot simply be another unconstrained generation step that comments on a prior generation. If a model generates both the idea and the judgment, the system may only be producing evaluative language. The architectural gap is whether evaluation is tied to evidence outside the immediate generation: prior decisions, reviewer outcomes, accepted and rejected directions, explicit researcher preferences, and later outcome feedback. Judgment becomes scientifically meaningful only when it can be audited against those traces and revised when its selections fail.

This concern deserves to be stated sharply, if the same model both generates a collision and evaluates it, the judgment layer risks collapsing into confirmation bias or stylistic mimicry of past acceptances. Four mechanisms guard against this circularity. First, judgment criteria are extracted from historical decision traces, reviewer outcomes, draft-to-final diffs, funded and rejected proposals, abandoned directions, which are fixed records that generation cannot alter. Second, generation and judgment should be architecturally separated, implemented as distinct models, or with judgment criteria frozen at inference time, so that the evaluator applies previously extracted standards rather than improvising evaluative language in the same pass. Third, ground-truth outcome feedback enters the layer through real selection events, whether a direction was subsequently pursued, funded, published, or abandoned provides labels that are external to any generation and that accumulate over time. Fourth, the blinded retrodiction and ranking evaluations described later in this section are designed to discriminate mimicry from alignment on held-out decisions, reproducing surface style is not sufficient to reproduce rankings, rejections, and rationales. Judgment, on this design, is auditable because its criteria, its evidence, and its failures are all externalized.

An explicit judgment layer also helps distinguish cognitive modeling from personalization or stylistic mimicry. A system that sounds like a researcher is not necessarily reasoning like one. The stronger test is whether it rejects bad ideas for the right reasons, recognizes when an apparently novel direction is actually trivial or infeasible, and prefers frontiers that are coherent with the researcher's accumulated trajectory. Judgment alignment is evidenced when the system and the researcher converge not only on which ideas to pursue, but on the reasons for pursuing them and the conditions under which they would revise, postpone, or abandon them. In this sense, rejection quality is as important as generation quality.

Early implementation experience also suggests that judgment should not be modeled only as positive ranking. A scientifically useful system must be able to abstain when evidence is too thin, identify framings that are attractive but weakly supported, and specify what should not be claimed from the available memory trace. In this sense, judgment is partly a theory of disciplined refusal. Rejection quality, scope-awareness, and overclaim control are not auxiliary safeguards layered onto generation after the fact; they are part of what makes generation scientifically credible in the first place.

Judgment also changes the role of novelty itself. In many generative systems, novelty is treated as the objective and feasibility or coherence as later constraints. Scientific reasoning works differently. Novelty is only one criterion among several, and often not the decisive one. An idea may be original yet strategically mistimed, insufficiently grounded, or methodologically fragile. Conversely, a direction that appears modest may be valuable because it consolidates a trajectory, answers a persistent critique, or opens a fundable line of inquiry. Judgment therefore does not merely filter generated ideas. It helps determine what counts as a good idea in the first place.

For this reason, the evaluation of judgment cannot be limited to output fluency or even to whether proposed studies appear reasonable to outside readers. It requires tests that probe alignment between the system's selections and the researcher's own reasoning. Such tests include retrodiction from a time-sliced archive, direct elicitation of divergent judgment through rankings and rationales over plausible frontiers, and a rejection benchmark that mixes plausible, trivial, incoherent, and infeasible ideas. Over time, accepted, rejected, deferred, and abandoned directions can themselves become evidence for judgment formation, creating a feedback loop in which the system learns not only from what was proposed, but from which proposals actually survived contact with real research constraints. These evaluations matter because the core claim is not that Mnemo should imitate style, but that it should help generate and sort possibilities in ways the researcher recognizes as rigorously and strategically plausible.

This framing also places an important limit on the ambition of the project. The goal is not to claim that all scientific judgment can be fully captured or exhaustively formalized. Some elements of judgment may remain tacit, unstable, or irreducibly contextual. But that does not make judgment unusable as an architectural concept. It means only that judgment must be treated as an empirical target: something to be approximated, tested, audited, and revised rather than presumed. What matters is that judgment is neither an optional extra nor a latent property that will automatically emerge from larger models and broader retrieval.

Seen in this light, differentiated memory and cross-memory collisions are necessary but not sufficient. They explain how a system can preserve continuity and generate structured novelty. Judgment explains how that novelty is filtered into a plausible research trajectory.

## 6 Mnemo as a research agenda and evaluation framework

Mnemo should not be presented as a finished autonomous scientist. It is better understood as a research agenda organized around falsifiable architectural claims. The central hypothesis is that AI systems for science may become more useful when they are grounded in differentiated memory, generate ideas through typed cross-memory collisions, and filter those ideas through a historically informed judgment layer. Framed this way, Mnemo is not primarily a product concept or a software stack. It is a program for asking what it would take to model scientific cognition in an operational and testable way.

This framing matters because the major components of the proposal are not all at the same stage of maturity. Some are largely engineering problems: provenance attribution, artifact identity, deduplication, memory routing, and evidence-linked retrieval. Others are research problems in a stronger sense: how to generate frontiers that are both non-trivial and grounded, how to extract stable judgment criteria from a researcher's history, and how to determine whether a system's proposals align with a real intellectual trajectory rather than merely fluent output. The immediate goal is therefore not to claim that a final exploration engine has already been specified, but to discover which mechanisms actually produce grounded, researcher-aligned novelty.

Within that agenda, typed cross-memory collisions are best treated as the current minimal testable generative mechanism. They are comparatively tractable to route and audit using existing provenance, retrieval, and ranking infrastructure. Richer dialogue-based or fully interactive models of scientific cognition should be treated as later extensions rather than pre-requisites for evaluating the framework's first architectural claims.

To be explicit, this Perspective does not present an implemented, end-to-end Mnemo system, and no claim made here depends on one. Preliminary internal prototyping should be read as formative design evidence rather than validation of a completed system. Early work tested whether differentiated memory, provenance-sensitive retrieval, and typed collisions could be operationalized over a private longitudinal research archive, but it did not establish general performance, autonomous scientific competence, or downstream research success. Operationally, the near-term unit is better understood as a Mnemo-based AI agent with a researcher in the loop. Structural memory organization and typed collision generation appear more directly operationalizable than paradigm-level reframing, which may still depend in part on general-purpose foundation models and researcher-guided judgment. This boundary does not weaken the framework; it clarifies which components are comparatively mature, which remain hybrid, and which require formal evaluation.

How, then, could Mnemo be implemented with current tools? Three pathways illustrate that the framework requires no exotic infrastructure and that its claims are testable with technology available today. The first is file-based differentiated memory within an agentic coding environment. Each memory space can be maintained as a set of structured, human-readable files with explicit provenance fields; routing policies can be expressed as agent instructions or skills that determine which spaces are consulted for a given task; collision operators can be orchestrated as typed prompts over records retrieved from two spaces; and judgment criteria can be encoded as further instruction files that constrain ranking and rejection. Our own formative prototyping used exactly this pattern, implemented over a commercial agentic environment (Claude Code, Anthropic), with the memory spaces represented as provenance-tagged document stores and routing enforced through explicit agent instructions. The second pathway is retrieval-based. A provenance-tagged vector index in which each record carries its memory-space assignment as first-class metadata, with routing implemented as typed filters over that metadata rather than as undifferentiated similarity search. The third is custom middleware between an archive and a foundation model, managing judgment extraction, outcome feedback, and audit trails, the components least served by current agent frameworks.

These pathways are not mutually exclusive; a mature implementation would likely combine file-based memory for inspectability, retrieval indexing for scale, and middleware for judgment and evaluation. What is architectural, and what any substrate must preserve, is not the storage technology but the typed provenance distinctions and the routing policies defined over them. In this sense, much of Mnemo can be understood as disciplined context engineering. The deliberate construction of what a model should be thinking with for a given task. The engineering is tractable; the open research questions concern what to route, when, and under which judgment criteria, not whether such routing can be built.

A useful evaluation framework must therefore operate at multiple levels. At the memory level, the system must distinguish authored work, external reference, critique, experience, and judgment-related material with high precision, preserve canonical intellectual artifacts across versions and linked documents, and retrieve evidence with correct provenance. At the routing level, it must show that different cognitive tasks draw on different memory configurations for principled reasons. At the generative level, it must show that typed collisions across memory spaces produce better candidate directions than flat retrieval or generic prompting alone. And at the judgment level, it must show that the system can rank, reject, abstain, and justify research directions in ways that converge with the researcher's own reasoning while resisting overclaim from thin evidence.

**Figure 3:**
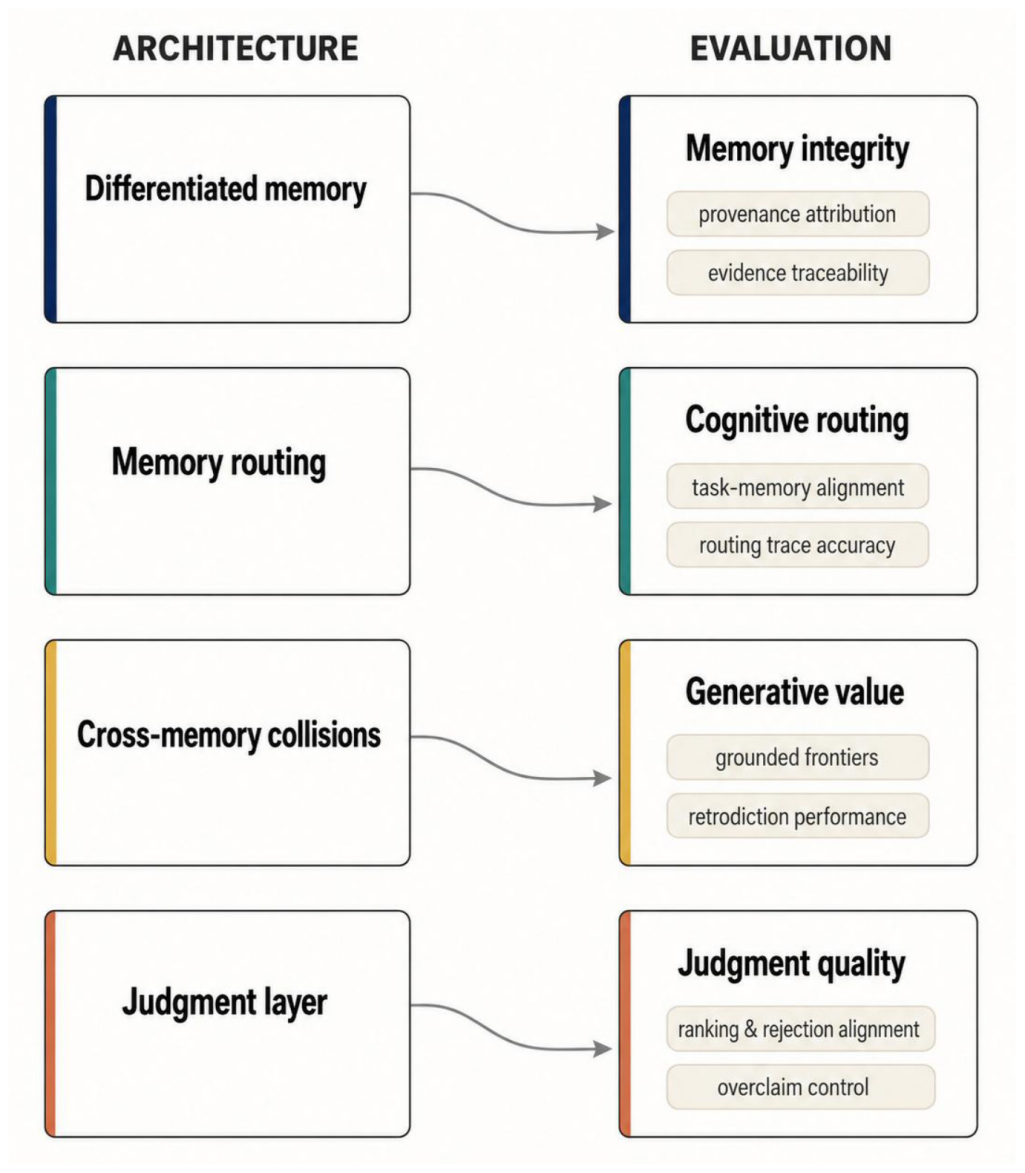
Evaluation domains aligned to architecture. The evaluation framework mirrors the proposed cognitive architecture: memory integrity (provenance, artifact identity, retrieval attribution, evidence traceability), cognitive routing (task-memory alignment and routing traces), generative value (grounded frontiers, retrodiction, baseline comparison), and judgment quality (ranking, rejection, abstention, rationale alignment, overclaim control). This architecture-to-evaluation mapping defines how Mnemo can be tested as a scientific-cognition framework rather than only as a workflow tool.

These requirements can be grouped into four evaluative domains. The first is **memory integrity**: provenance attribution, artifact identity, retrieval attribution, and evidence traceability. The second is **cognitive routing**: whether task-specific memory selection is accurate, inspectable, and defensible. The third is **generative value**: whether cross-memory collisions produce grounded, non-trivial frontiers that outperform flat retrieval or generic baselines. The fourth is **judgment quality**: whether the system ranks, rejects, abstains, and justifies candidate directions in ways that align with the researcher's own rationales while preserving calibration between evidence and speculation and maintaining overclaim control. Figure 3 maps these evaluative domains back onto the architecture they are intended to test.

Within this broader architecture, different experiments can probe different parts of the framework. Memory-oriented tests can evaluate provenance classification, retrieval attribution, and routing accuracy. Generative tests can examine whether the system can surface meaningful links across seemingly unrelated projects, retrodict future directions from earlier slices of an archive, and produce better ideas than generic baselines. Judgment-oriented tests can compare the system's rankings, rationales, and rejection logic with the researcher's own choices under blinded conditions. The point is not that any single benchmark will settle the question. It is that the framework becomes scientifically useful only when its components can fail in interpretable ways.

A further extension becomes visible once the framework is implemented beyond a static archive. Scientific cognition is not only retrospective. It is also shaped by active projects, emerging collaborations, student-led pivots, abandoned directions, and the outcomes of real selection events. This extension is best understood not as a sixth memory space, but as an operational layer linking the five memory spaces to live project state and outcome feedback. A mature version of the framework would therefore connect longitudinal archive memory to current project activity and selection outcomes, allowing the system to learn not only from what a researcher has done, but from which directions are currently being pursued, rejected, or transformed in real time. One longer-term extension would be to couple this live-state layer to mediated simulation environments such as digital twins or XR-based analytic interfaces (Dawkins and Kitchin, 2025; Antoun et al., 2025). In the present paper, however, such environments should be treated as future implementations of live-state coupling rather than as part of Mnemo's core claim about provenance-routed research memory.

This is also where the framework becomes broader than a single case study. Mnemo is motivated here by one researcher's archive and one longitudinal intellectual history, but the architecture is not meant to be idiosyncratic to that case. The claim is not that all scientists reason identically. It is that sustained scientific work generally depends on differentiating one's own prior commitments from external references, learning from critique, drawing from experience beyond formal publications, and developing recurrent patterns of judgment about what is worth doing next. For that reason, the core architecture is potentially domain-independent even if its specific memory contents, judgment criteria, and frontier structure are not. Generalizability, however, should be treated as an empirical question rather than an assumption.

The larger implication is that progress in AI for science should not be assessed only by how much of the workflow can be automated or how persuasive generated prose appears. The stronger standard is whether such systems help researchers think better, faster, and more strategically without losing rigor or intellectual identity. That shifts evaluation from throughput to grounded scientific reasoning. Mnemo is offered in that spirit: not as a claim that scientific cognition has been solved, but as a framework for studying how it might be modeled, tested, and improved.

## 7 Conclusion

The recent rise of AI research agents has shifted the conversation from whether parts of science can be automated to how far that automation can extend. Systems such as The AI Scientist show that large portions of the research workflow can already be coordinated by foundation models and agentic scaffolds. That achievement is substantive. The next advance in AI for science, however, will require a shift from pipelines that execute tasks to architectures that preserve intellectual continuity, learn from critique, and exercise judgment over possible futures.

We have argued that differentiated memory provides a candidate minimal architecture for this shift. On this account, scientific reasoning may depend on maintaining distinctions between authored work, external reference, critique, experience, and accumulated judgment, because these are not merely different sources of text, but different kinds of memory with different cognitive roles. In that sense, provenance functions as memory routing.

We have further argued that differentiated memory may become generative through controlled collisions across memory spaces. Scientific novelty is not well described as random recombination or generic one-shot ideation, and may be better understood as arising from structured interactions among continuity, adjacency, contradiction, transfer, and pressure. Yet generation alone is insufficient. A cognitive system for science must also model judgment: the historically accumulated criteria by which a researcher recognizes some possibilities as worth pursuing and rejects others as trivial, incoherent, mistimed, or infeasible.

Mnemo is therefore not offered as a claim that scientific cognition has already been solved, nor as a finished autonomous scientist. It is offered as a research agenda and evaluation framework. In the near term, such systems may function less as autonomous replacements for scientific reasoning than as structured cognitive partners: preserving differentiated memory, surfacing grounded collisions, and supporting judgment without eliminating the role of human reframing and selection. Its value will depend on whether systems built from these ideas can demonstrate strong memory integrity, interpretable routing, productive use of critique, trajectory-grounded judgment, and clear separation between evidence and speculation.

The broader implication is that progress in AI for science should not be judged only by how much of the workflow can be automated or how persuasive generated outputs appear. The stronger standard is whether such systems help researchers think in ways that remain grounded, cumulative, selective, and strategically coherent over time. The question is no longer only whether AI can produce research artifacts. It is whether AI systems can participate in inquiry without losing the memory structure and judgment that make scientific reasoning recognizably scientific.

## Data Availability

The original contributions presented in the study are included in the article/supplementary material, further inquiries can be directed to the corresponding author.
